# Adherence to anti-diabetic drugs among patients with Type 2 diabetes mellitus at Muhimbili National Hospital, Dar es Salaam, Tanzania- A cross-sectional study

**DOI:** 10.11604/pamj.2014.17.252.2972

**Published:** 2014-04-07

**Authors:** Godfrey Mutashambara Rwegerera

**Affiliations:** 1 University of Botswana, School of Medicine, Gaborone, Botswana

**Keywords:** Type 2 diabetes mellitus, anti-diabetic drugs, drug adherence, Tanzania

## Abstract

**Introduction:**

Adherence to diabetes mellitus treatment regimens among Type 2 diabetes patients in Tanzania has not been well documented. This study sought to assess adherence to antidiabetic drugs and associated factors among patients with Type 2 diabetes mellitus.

**Methods:**

A cross-sectional study was conducted among type 2 diabetes mellitus patients who were attending the Diabetic clinic of Muhimbili National hospital between May 2009 and February 2010. Assement ofadherence to antidiabetic medications was based on patients’ self-reported recall of skipped days without taking medications, over the past one week and three months. Data wereentered and analyzed using Statistical Package for Social Sciences (SPSS Inc. Chicago, Illinois version 16). The crude and adjusted odds ratio (COR/ AOR) and 95% Confidence Interval (CI) were performed to determine factors associated with anti-diabetic medications adherence and a p-value of 0.05 or less was considered statistically significant.

**Results:**

Adherence rates to antidiabetic drugs were found to be 60.2% and 71.2% at one week and three months respectively. High cost of medication was significantly associated with anti-diabetic non-adherence. Adherence to anti-diabetic drugs significantly increased with an increase in number of non-diabetic medications.

**Conclusion:**

Adherence to antidiabetic drugs was found to be suboptimal. Patients with other medical conditions in addition to diabetes mellitus are more likely to adhere to anti-diabetic medications. There is a need for the responsible authorities to set policies that subsidize cost of anti-diabetic drugs to improve adherence and reduce associated complications.

## Introduction

Diabetes mellitus is a significant and growing health problem worldwide. It is a chronic disease that requires long-term medical attention both to limit the development of its devastating complications and to manage them when they do occur. Diabetes mellitus was estimated to affect at least 285 million people worldwide in 2010, and the number is expected to reach 438 million by the year 2030[1].

Diabetes mellitus (DM) is also an important problem in Africa. In Sub-Saharan Africa, like the rest of the world, diabetes prevalence coupled with both communicable and non-communicable diseases is on the rise[2]. According to International Diabetes Federation, it was estimated that by 2010, about 12.1 million people were living with diabetes in Africa, and the number is projected to increase to 23.9 million by 2030[3].

The burden associated with diabetes is enormous in terms of increased morbidity and mortality and the economic strains associated with the disease. Poorly managed patients have poor quality of life, have prolonged and frequent hospital admissions. All these impact on the patients and the family members who have to take care of them. They also impact on the health care system, the government and the society as a whole.

In 1996, McLarty et al reported diabetes to be an important cause of adult mortality in Tanzania, reaching mortality rates comparable to those reported among diabetic patients in the USA [4].

An investment in diabetes care brings health gains in other disease areas such as hypertension and coronary artery diseases. Low cost strategies such as lifestyle modification, increasing physical activity and effective drug use have been shown to reduce the impact of diabetes and other associated diseases [5].

Previous studies in Mexico, Jamaica, United States of America and India which were conducted between 1999 and 2002 respectively found adherence to diabetes treatment generally to be sub-optimal ranging from 23 to 77% [6–10]. In another study among patients with type 2 diabetesmellitus, adherence was found to range between 65% to 85% for oral agents and 60% to 80% for insulin [11].

Despite compelling evidence about the effectiveness of medications, adherence to treatment has been recognized to be a major problem in patients with chronic illness [12, 13, 14], and a large proportion of patients become non -adherent at six months from treatment initiation [12, 15].

Rates of non-adherence with any long term medication treatment vary from 17% to 60%, depending on the characteristics of the condition, the treatment, the patient, and the setting [16, 17]. Of significance, non-adherence is the highest when the patients are symptom –free [17].

Vermeire E et al showed factors including regimen complexity (number of medications and number of doses per day), cost and side effects to be associated with diabetic non-adherence [18]. Patients who did not understand their drug regimens well had higher risk of non-adherence than those who understood them well [11, 18, 19]. Other studies conducted on regimen complexity as a reason for non-adherence did not show significant association [20, 21].

Drug non-adherence has been previously demonstrated with advancing age; this was attributed to the fact that, advanced age is associated with poor/decrease cognition and increased number of drugs due to several comorbidities [5].

In a study conducted in Mulago hospital in Uganda, almost one third of respondents (31.3%) in the age group 36 to 50 years were not adhering, however overall age was not a significant factor affecting adherence. Women had almost threefold increase in risk of non-adherence as compared to men. Results of this study further revealed gender to be significantly associated with drug non-adherence [19]. In a study performed in Brazil, adherence to anti-diabetic medications improved with increase in level of education, however, the association was not significant [22].

Several studieshave reported that patients with multiple medical conditions associated with increased age such hypertension, hyperlipidemia and acute coronary syndromes actively choose which treatments to forego when cost pressuresbecome a problem [23, 24].

The study conducted in Ibadan, Nigeria revealed that 59% of patients were non-adherent with the previous anti-diabetic drugs due to lack of finance (51.7%). The study further revealed that 34.5% of patients were non-adherent with the previous anti-diabetic drugs due to side effects [25]. Side effects, as a reason for non-adherence was also the most attributing factor in another study [11].

Depression has also been identified to be a significant factor challenging DM medication adherence, with depressed patients less likely to report and demonstrate good adherence (42% of depressed patients reporting good adherence versus 67% non-depressed patients) [26]. Another study on the effects of depression on DM identified medication non-adherence rates of 24.5% for those with depression versus 18.8% for those with no major depression [27].

Furthermore, it was revealed in the study conducted in Uganda that, patients who took longer time to visit a health worker were more likely not to adhere [19].

Results from different studies revealed other socio-demographic factors like marital status, occupation of the respondent and religion were not significantly associated with non-adherence [6, 13, 19, and 21]. Also other studies revealed other factors like duration of diabetes, route of administration and number of anti-diabetic drugs prescribed were not significantly associated with non-adherence [19–21].

Recently, it was found in ENTRED study, a large population study among patients with type 2 diabetes in Europe on overall medication adherence including anti-diabetic drugs;-that factors including age, financial difficulties and existing diabetes complications were associated with poor medication adherence [14].

There is paucity of information on medication adherence in patients with type 2 diabetes mellitus in Tanzania. Thus, this study was conducted to assess the extent of anti-diabetic drug adherence and determine factors associated with non-adherence among patients with type 2 diabetes mellitus.

## Methods

A descriptive cross-sectional study was conducted at the Diabetic clinic of Muhimbili National Hospital, the largest consultant and teaching hospital in Tanzania. The clinic saves for patients within Dar es Salaam and referrals from around the country. The study was conducted over a period of nine months starting from May 2009.

### Participants

The study included patients who had type 2 diabetes mellitus, based on documentation in their medical files and/or the use of oral hypoglycemic medications. Patients were included if they had been on drug treatment for diabetes mellitus for a least three months at the time of enrolment. Patients with type 1 diabetes mellitus, pregnant women and patients with mental illnesses who could not consent were excluded. On a clinic day, a list of all attending patients was made. A random selection was used to select the first patient. Subsequent recruitment involved every 5^th^ patient following each enrolment. Informed written consent was obtained from all the patients who agreed to participate, in cases of refusal; the 7^th^ patient was enrolled without affecting the order.

### Sample size determination

Sample size was calculated from the formula of descriptive cross-sectional study:

N = Z^2^ p (100-p)/d^2^

Where:

N = Estimated minimum sample size

Z = Standard deviation of 1.96 at 95% confidence interval

P= Prevalence of adherence among diabetic patients at Mulago hospital in Uganda, estimated to be less than 72%

d = margin of error on p, approximately 0.06

Hence, N= 1.96x 1.96x72x28/6^2^

N = 215

**Data collection**: using structured questionnaires, patients’ age, sex, date of first diagnosis, previous and current anti-diabetics and any other medications were recorded.

**Assessment of adherence**: Adherence to medications was assessed based on patients’ recall on the use of anti-diabeticdrugs in the previous one week and three months. Good adherence was considered if the patient's calculated adherence was at least 80% of the expected days. Patients with less than 80% days of using their medications were judged non-adherent (poor adherence). The cut-off point of 80% was considered acceptable as it was also used in other studies of chronic medications [28, 29]. For patients using more than one anti-diabetic drug, the worst computed adherence of the drugs was used to represent the patient's overall adherence.

**Clinical evaluation**: Patients’ weight and height were measured and used to calculate the Body Mass Index (BMI) as a ratio of weight in kilograms/height in meters squared [30]. Peripheral neuropathy was assessed by 5.07/10g- Semmens Weinstein monofilament testingand classified as either significant neuropathy or normal perception to non-significantneuropathy [31, 32]. Peripheral vascular disease assessment was according to Ankle brachial plexus index (ABPI) using Dopplerultrasound [33]. Patients’ mean blood pressure was recorded during evaluation and hypertension defined according to Joint National Committee (JNC 7)[34].

**Glycemic control:** Patients’ glycaemic control was assessed using fasting blood glucose/random bloodglucose and glycosylated haemoglobin (HbA1c). Measurement of HbA1c isnot a routine practice; - it was measured in only 62 patients who were randomly selected on each clinic visit. Fasting blood glucose (FBG) or random bloodglucose levels (RBG) were determined in all our patients. Good glycemic control wasdefinedas HbA1cl of < 6.5% to 7.0%. Levels of HbA1c between 7.1 to 7.5% were defined as satisfactory glycemic control. Patientswith poor glycemic control were those whoseHbA1c levels were above7.5% or had fasting blood glucose of more than 6.1 mmol/l or random blood glucose 2-hours after a meal of more than 7.1mmol/l. [35–37].

**Statistical analysis:** The data were analyzed using the statistical Package for Social Sciences (SPSS Inc. Chicago, Illinois) software, version 16. Frequencies and descriptive statistics were computed for the variables. The association between variables and adherence was analyzed by cross-tabulation, and the significances were tested by the chi-square and Fisher's exact test. The crude and adjusted odds ratio (COR/ AOR) and 95% Confidence Interval (CI)were performed to determine factors associated with anti-diabetic medications adherence and a p-value of 0.05 or less was considered statistically significant. The study was approved by the Ethical Review Board of Muhimbili University of Health and Allied Sciences

## Results

**Characteristics of study participants**: The study included a total of 216 diabetic patients, with a median age of 55.0 years (34-81 years). Two-thirds of studied patients were females. About half of the patients (51.4%, n= 111) had primary education. Most of the patients (62.5%, n=135) had diabetes for more than 5 years. The majority (72.7%, n= 157) were either overweight or obese (Table 1)

**Table 1:** Characteristics of study patients with type 2 diabetes mellitus and associated complications

Demographic factor	N= 216	%
**Age (years)**		
<40	21	9.7%
41-50	48	22.2%
51-60	74	34.3%
>60	73	33.8%
**Gender**		
Male	72	33.3%
Female	144	66.7%
**Religion**		
Christian	*110*	50.9%
Moslem	106	49.1%
**Education level**		
No formal education	26	12%
Primary education	111	51.4%
Secondary education	48	22.2%
Post-secondary education	31	14.4%
Duration of diabetes (years)*		
< 2	38	17.6%
2-5	43	19.9%
> 5	135	62.5%
Body Mass Index**		
Underweight	4	1.9%
Normal	52	24.1%
Overweight	87	40.3%
Obesity	70	32.4%
**Peripheral vascular disease (Doppler findings)**		
Normal	192	88.9%
Abnormal	24	11.1%
**Peripheral neuropathy (monofilament testing)**		
Normal perceptionplus non-significant neuropathy	143	66.2%
Significant neuropathy	73	33.8%
**Hypertension**		
Normal (< 140/90mmHg)	113	52.3%
High BP (≥ 140/90mmHg)	103	47.7%

* Median duration (range), 7 years (0.3-34 years)

** 3 patients were not as assessed for nutritional status due to inconvenience of measuring either their weight or height.

### Pattern of adherence to anti-diabetic drugs

Good adherence to diabetes medications was reported in 60.2% and 71.2% at one week and three months respectively. (Table 1). Drug adherence was better among patients older than 61 years (71.2%). Patients in the age group of 41-50 years had the poorest adherence (43.8%). Similarly; concurrent use of non-diabetic medications was significantly associated with good adherence. Gender, religion, level of education, duration of diabetes and number of oral hypoglycemic agentsdid not significantly affect adherence to anti-diabeticmedications. (Table 2). In multivariate logistic regression, increase in the number of drugs other than antidiabetics significantly increased anti-diabetic medication adherence (Table 2)

**Table 2:** Association between anti-diabetic drug adherence and characteristics of patients with type 2 diabetes mellitus

Characteristics of patients	Adherence in previous week*		Crude OR (95% Confidence interval	Adjusted OR (95% Confidence interval
	Good N(%)	Poor N(%)		
**Age (years)**				
< 40	14(66.7%)	7(33.3%)	Reference	
41-50	21(43.8%)	27(56.2%)	0.389(0.133,1.136)	
51-60	43(59.1%)	29(40.9%)	0.694(0.251,1.920)	
>61	52(71.2%)	21(28.8%)	1.238(0.438,3.500)	
**Gender**				
Male	47(65.3%)	25(34.7%)	Reference	
Female	83(57.6%)	61(42.4%)	0.724(0.402,1.302)	
**Religion**				
Christian	61(55.5%)	49(44.5%)	Reference	
Muslim	69(65.1%)	37(34.9%)	1.498(0.866,2.593)	
**Education level**				
No formal education	15(57.7%)	11(42.3%)	Reference	
Primary education	68(61.3%)	43(38.7%)	1.160(0.487,2.759)	
Secondary education	30(62.5%)	18(37.5%)	1.222(0.462,3.234)	
Post-secondary education	17(54.8%)	14(45.2%)	0.890(0.311,2549)	
**Duration diabetes (years)**				
< 2	24(63.2%)	14(36.8%)	Reference	
2-5	25(58.1%)	18(41.9%)	0.810(0.331,1.983)	
> 5	80(59.7%)	54(40.3%)	0.875(0.416,1.841)	
**Number of anti-diabetics**				
1	55(65.5%)	29(34.5%)	0.736(0.416,1.304)	
> 1 (2 or 3)	75(56.8%)	57(43.2%)	0.132(0.014,1.234)	
Other drugs**				
Antidiabetic alone	54(49.5%)	55(50.5%)	Reference	Reference
Antidiabetic + 1	33(68.8%)	15(31.2%)	2.241(1.094,4.588)	2.156(1.017,4.570)
Antidiabetic +2	19(67.9%)	9(32.1%)	2.150(0.894,5.171)	1.815(0.712,4.6240
Antidiabetic +3 or more	24(77.4%)	7(22.6%)	3.492(1.389,8.779)	3.095(1.182,8.106)
**Peripheral vascular disease(Doppler findings)**				
**Normal**	114(59.4%)	78(40.6%)	Reference	
**Abnormal**	16(66.7%)	8(33.3%)	1.368(0.558,3.353)	
**Peripheral neuropathy**				
Normal perception plus non-significant neuropathy	90(62.9%)	53(37.1%)	Reference	
Significant neuropathy	40(54.8%)	33(45.2%)	1.401(0.790-2.433)	
Blood pressure**				
Normal (<140/90mmHg)	67(59.3%)	46(40.7%)	Reference	
High Blood pressure (≥140/90mmHg)	63(61.2%)	40(38.8%)	1.866(1.018,3.420)	1.333(0.683,2.603)

Good = adherence of ≥ 80%, Poor = adherence <80%; Antidiabetic+ indicate antidiabetic drug plus 1/2/3 other non-diabetic drugs

** Multiple logistic regression analysis was made for selected variables with p-value <0.05 (statistical analysis in this case was good adherence versus poor adherence)

Peripheral vascular disease and peripheral neuropathy were the complications analyzed against anti-diabetic medication adherence. They were found to have no significant association with medications adherence.

Hypertension, a common comorbid condition in patients with type 2 diabetes mellitus was significantly associated with good anti-diabetic medications adherence in univariate analysis, however there was no significant association when multivariate logistic regression was performed (Table 2).

### Reasons for anti-diabetic medicationsnon-adherence

Patients with poor adherence reported several reasons for them not adhering to anti-diabetic medications. High cost of antidiabetic drugs (52.7%; n=78), disappearance of symptoms (18.9%; n=28), drug side effects including fainting, fatigue, palpitations, nausea, vomiting and itching (11.49%, n=17) were the main reasons for non- adherence. Other reasons for non-adherence were the use of traditional medicine, forgetting and alcohol use (Table 2).

Metformin was the most used drug, either as a single drug or in combination, its use accounted for 154(71.3%), Glibenclamide was the second most commonly used drug, i.e. 120(55.6%), while 33(15.3%) of the patients were on Insulin injections (Table 3)

**Table 3:** Reasons for non-adhering to anti-diabetic medications

Adherence	B	S.E	Significance	Exp(B)	95% CI for EXP(B)
Reasons for missing drug 1*					
High cost	-1.300	0369	0.000	0.272	0.132-0.562
Constant***	0.566	0.273	0.038	1.762	
Reasons for missing drug 2*					
High cost	-2.083	0.489	0.000	0.125	0.048-0.325
Constant***	1.605	0.367	0.004	2.900	
Reasons for missing drug 3**					
High cost	-1.609	1.483	0.278	0.200	0.011-3.661
Constant***	0.916	0.837	0.273	2.500	

* Drug 1 and 2 refers to any of Metformin, Glibenclamide, Chlropropamide or Insulin analyzed separately.

** Drug 3 refers to the rare used drugs including Glipizide, Gliclazide, Rosiglitazone and Pioglitazone.

*** Constant refers to all other reasons than cost combined.

Seventy seven patients, 77(35.6%) were on single antidiabetic treatment, 134 (62.0%) were on two antidiabetic drugs while 5 (2.4%) were on three anti-diabetic drugs. None of the studied patients was on a combination of oral hypoglycemic agent and Insulin.

The effect of high cost ofindividual most commonly used drugs namely Metformin, Glibenclamide, Chlropropamide and Insulin was compared to all other reasons for non-adherence combined. Binary logistic regression analysis showed high cost of medication to be significantly associated with non-adherence (p< 0.05) (Table 3)

**Anti-diabetic medication adherence and glycemic control:** the mean glycosylated haemoglobin (HbA1c)in a subset of 62 patients was 9.42%. Good glycemic control usingHbA1c and fasting/randomblood glucose was found in 15/62(24.2%) and71/216(32.9%) of study participants respectively. Good anti-diabetic medication adherence wasassociated with better glycemic controlusing HbA1c (73.3%for good adherence versus 66.0% for patients with poor adherence). This was also true for glycemic control using fasting/random blood glucose (69.0% for good adherence versus 55.9% for patients with poor adherence), however the differences were not statistically significant.

## Discussion

The prevalence of anti-diabetic drug adherence found in this study was suboptimal both at one week and three months. Since adherence information was based on patients’ recall, the actual prevalence may be even lower than that was found in our study. Nevertheless, similar rates of adherence have been reported in studies done in India, Jamaica, Uganda and France [7, 10, 14, 19].

Adherence to treatment in this study differed between three months and one week. This may be due to the fact that it easier to recall the number skipped days (without medications) at one week than within the past three months.

In previous studies, several factors were inconsistently associated with poor adherence to anti-diabetic treatment. They include regimen complexity, cost and side effects of medications, advanced age, female gender, long duration of diabetes, and comorbid conditions such as hypertension, hyperlipidemia, coronary artery disease and depression [11, 14, 18, 19, 23–26]. In this study, good adherence was found among elderly patients. This positive effect of age on adherence was alsoreported in the United Kingdom [38]. In the current study; patients in the middle age group of 41-50 years had the poorest adherence. This is comparable to the findings of the study done in Uganda where majority of non-adherent patients were in the age group 36-50 years [19]. In this study gender was not associated with adherence, however; -other studies found female gender to be associated with poor anti-diabetic medication adherence. This could be explained by the fact that women are more prone to stress and to develop mental and emotional disorders like depression, the factors not assessed in the current study. Another interesting factor that was associated with adherence was the concurrent use of non-diabetic medications with anti-diabetic drugs. The use of other medications in addition to anti-diabetic drugs was significantly associated with a good adherence. These patients are likely to have multiple cormobidies, attend different clinics and hence more information on the benefits of compliance to medications. Multiple comorbidities are also likely to occur with aging. Thus, age-related improved adherence may partly be explained by increased cormobidities [17]. Similar to a study done in the United States of America, there was no association between gender and adherence to medications [8].

Other social demographic characteristics analyzed were level of education and religion;-they were not associated with anti-diabetic drug adherence. This finding is similar to that of other studies, where, religion was not associated with adherence [8, 13, 19].

Likewise, the duration of diabetes and the number of anti-diabetic drugs used had no significant association with degree of anti-diabeticmedications adherence. Similar findings have also been observed in other studies [8, 13, 19, 22]

Majority of patients in this study were either overweight or obese, the two accounted for 72.7% of all patients. Being obese or overweight has been found to have a negative influence on adherence resulting to patients not following dietary advice or fear of weight gain associated with medication use [39]. Other studies have shown patients with diabetes mellitus to have depression twice as much as in matched controls without diabetes, and more research evidence has associated depression with decreased physical activity, hence higher overweight and obesity rates in diabetes, and also depression was associated with poor adherence to antidiabetic medication [26, 40]. Depression was not assessed in this study; it could have a bearing effect on the pattern of anti-diabetic medications adherence.

Failure to afford medications was the most common reason for poor adherence. The hospital provides free medications for only one month. As appointments are always longer than a month apart, most of the patients are forced to refill their prescriptions from private pharmacies. This is only possible for few patients who can afford. Majority of the patients may have to wait until the scheduled visit to get free medication. Nevertheless, similar findings have been reported elsewhere [11, 25].

Drug side effect was the other reason associated with poor adherence. In contrast to the Ibadan study where drug side effects accounted for about 34.5% of non-adherence, only 11.6% of our patients reported drug side effects as their reason for non-adherence [25].

In this study, patients who had good glycemic control had better adherence to anti-diabetic drugs compared to those who had poor glycemic control, however; -this was not statistically significant. The lack of association between anti-diabetic drug adherence and glycemic control was also seen in other studies [41–43].

Other studies of self-reported adherence have also shown lack of significant association between anti-diabetic drug adherence, glycemic control and complications of diabetes. One study done in United States of America revealed glycemic control and high blood pressure were not associated with drug adherence, possible explanation for lack of association was that;- either patients were over- reporting their adherence or doctors were not prescribing sufficient potent doses to achieve glycemic control [44], these explanations could also account for the situation observed in this study.

This study had several limitations;-firstly, the sample size was calculated based on adherence studies, a larger sample size would have sufficed to provide a better relationship on association between anti-diabetic medicationsadherence versus complications/comorbidconditions.

On the other hand, use of self-report method to assess adherence to anti-diabetic drug therapy is associated with recall bias;-there is a tendency to overestimate adherence. This modality of assessing adherence has its benefits, as it helps clinicians to identify patients who are more likely to have poor adherence, and determine aspects of diabetes that they can focus to improve patients’ management [45].

There was an effort to determine association between anti-diabetic medications adherence and glycemic control using glycosylated haemoglobin(HbA1c). Due to financial constraints, HbA1c was performed to only about a quarter of study participants.

Finally, management of diabetic mellitus has three core components namely dietary adherence, regular physical activity and appropriate drug therapy coupled with regular self-monitoring of blood glucose by patients, this study did not address some of these factors.

## Conclusion

Adherence to anti-diabetic drugs was found to be suboptimal. High cost of medications was significantly associated with poor anti-diabetic drug adherence calling for responsible authorities to set policies that subsidize cost of anti-diabetic medications to improve adherence and associated complications.
